# Trends in Parent-Child Correlations of Childhood Body Mass Index during the Development of the Obesity Epidemic

**DOI:** 10.1371/journal.pone.0109932

**Published:** 2014-10-17

**Authors:** Teresa A. Ajslev, Lars Ängquist, Karri Silventoinen, Jennifer L. Baker, Thorkild I. A. Sørensen

**Affiliations:** 1 Institute of Preventive Medicine, Frederiksberg and Bispebjerg Hospital, Frederiksberg, Denmark; 2 Novo Nordisk Foundation Center for Basic Metabolic Research, Faculty of Health and Medical Sciences University of Copenhagen, Copenhagen, Denmark; 3 Population Research Unit, Department of Social Research, University of Helsinki, Helsinki, Finland; McMaster University, Canada

## Abstract

**Background:**

The intergenerational resemblance in body mass index may have increased during the development of the obesity epidemic due to changes in environment and/or expression of genetic predisposition.

**Objectives:**

This study investigates trends in intergenerational correlations of childhood body mass index (BMI; kg/m^2^) during the emergence of the obesity epidemic.

**Methods:**

The study population was derived from the Copenhagen School Health Records Register, which includes height and weight measurements since birth year 1930. Mothers and fathers with BMIs available at ages 7 (n = 25,923 and n = 20,972) or 13 years (n = 26,750 and n = 21,397), respectively, were linked through the civil registration system introduced in 1968 to their children with BMIs available at age 7 years. Age- and sex-specific BMI z-scores were calculated. Correlations were estimated across eight intervals of child birth years (1952–1989) separately by sex. Trends in these correlations were examined. Whereas the mother-child correlations reflected the biological relationship, a likely decline in the assignment of non-biological fathers through the registration system across time must be considered when interpreting the father-child correlations.

**Results:**

The BMI correlations between mothers and sons ranged from 0.29–0.36 and they decreased marginally, albeit significantly across time at ages 7–7 years (−0.002/year, p = 0.006), whereas those at 13–7 years remained stable (<0.0004/year, p = 0.96). Mother-daughter correlations ranged from 0.30–0.34, and they were stable at ages 7–7 years (0.0001/year, p = 0.84) and at 13–7 years (0.0004/year, p = 0.56). In contrast, father-son correlations increased significantly during this period, both at ages 7–7 (0.002/year, p = 0.007) and at ages 13–7 years (0.003/year, p<0.001), whereas the increase in father-daughter correlations were insignificant both at ages 7–7 (0.001/year, p = 0.37) and at ages 13–7 years (0.001/year, p = 0.18).

**Conclusion:**

During the obesity epidemics development, the intergenerational resemblance with mothers remained stable, whereas the father-child BMI resemblance increased, possibly reflecting changes in family relationships, and unlikely to have influenced the epidemic.

## Introduction

Children resemble their adult parents in body mass index (BMI = weight/height-squared; kg/m^2^) both during childhood and as adults [1]–[9]. Studies of individuals adopted-away early in life [10], [11] and studies of twins [12], [13] show that this resemblance is due mainly to genetic similarities between relatives. The environment shared by family members apparently has little or no influence on the BMI, perhaps except for some influence when the children still live with their parents in the same household [13].

The obesity epidemic is obviously due to major changes in the environmental determinants either directly or indirectly involved in obesity, but it remains unclear whether this has implied changes in the familial resemblance. The prevailing understanding of the causes of the obesity epidemic is that it is due to societal changes in the so-called obesogenic environment that primarily affect those genetically predisposed to obesity. If so, it would be expected that the familial resemblance in the underlying trait, BMI, would increase during the development of the obesity epidemic. On the other hand, influences from the obesogenic environment independent of the genetic predisposition would lead to less familial resemblance. Previous studies of correlations in BMI between brothers and among twins do suggest that the expression of the genetic predisposition and hence the familial resemblance is greater the greater the obesity prevalence [14]–[16].

In Denmark, the obesity epidemic among children and young men developed in phases, alternating between periods of stable and periods of increasing prevalence of obesity [17]–[19]. The phase-wise development was closely related to birth years, with an increasing prevalence of obesity observed in children born after the early 1940s and after the early 1970s. This opens for the possibility that corresponding changes in BMI during childhood in the parental generation may have influenced BMI in the child generation, which could be manifest in phase-wise changes in parent-child correlations during the development of the obesity epidemic.

Another reason for changes in the parent-child correlations during the epidemic could be changes in the degree of assortative mating related to BMI, possibly leading to clustering of genetic and environmental risk factors within families. In the above mentioned Danish population, assortative marriages did in fact occur among those who were the heaviest in childhood, and the tendency increased during the emergence of the obesity epidemic [20].

Using the same Danish population, we investigated the secular trends in the parent-child correlations of BMI, measured during childhood in both generations, across the birth years of the emergence of the obesity epidemic. The hypothesis was that the parent-child correlations would increase during the development of the obesity epidemic possibly due to assortative mating as well as greater expression of genes constituting the genetic predisposition.

## Materials and Methods

The study population was derived from the Copenhagen School Health Records Register (CSHRR) [21]. The original CSHRR register includes information on height and weight routinely measured by school health doctors or nurses on 372,636 children who underwent health examinations in public and private schools in the Copenhagen municipality, annually until 1984 and thereafter at school entrance and exit [21].

All citizens alive on April 2, 1968 and onwards were assigned a personal identification number. Children who were in school at this time or after, had the number recorded on their health card, and for the other children the number was retrieved to the extent possible (the main reasons for missing identification being death and emigration before the introduction of the number). Thus, these numbers were identified in ∼89% of all children in the register.

Family connections between parents and their biological children were identified by record linkage to the nation-wide so-called Fertility Database, which was established by Statistics Denmark in 1980 and updated since then [22]. Personal identification numbers were assigned to all Danish Citizens in 1968. Nationwide population counts were performed by the municipalities in 1970 and 1981 in which family unit information were kept. The last manual population count was performed in 1981. All children under the age of 26 years living at home were connected with their parents through the identification numbers, which have subsequently been computerized into the Fertility Database.

The procedure used for establishing the family units was stepwise built up from 1968 and onwards. This means that some of the assigned parents were not the biological parents because of preceding exchanges of the spouses or partners, and the proportion of families in which this may have happened is expected to be greater the older the children in the families were during the process of building the family units. Since the children usually stayed with the mother, this problem is presumed to be particularly relevant for the father-child relationships, and much less so for the mother-child relationships. Throughout the study period, there is of course a risk of actual non-paternity, which, however, is presumed to much less than the problem of exchanges of spouses assigned to be non-biological fathers. The implication for the interpretation of the father-child correlations for children is addressed in discussion.

**Figure 1:**
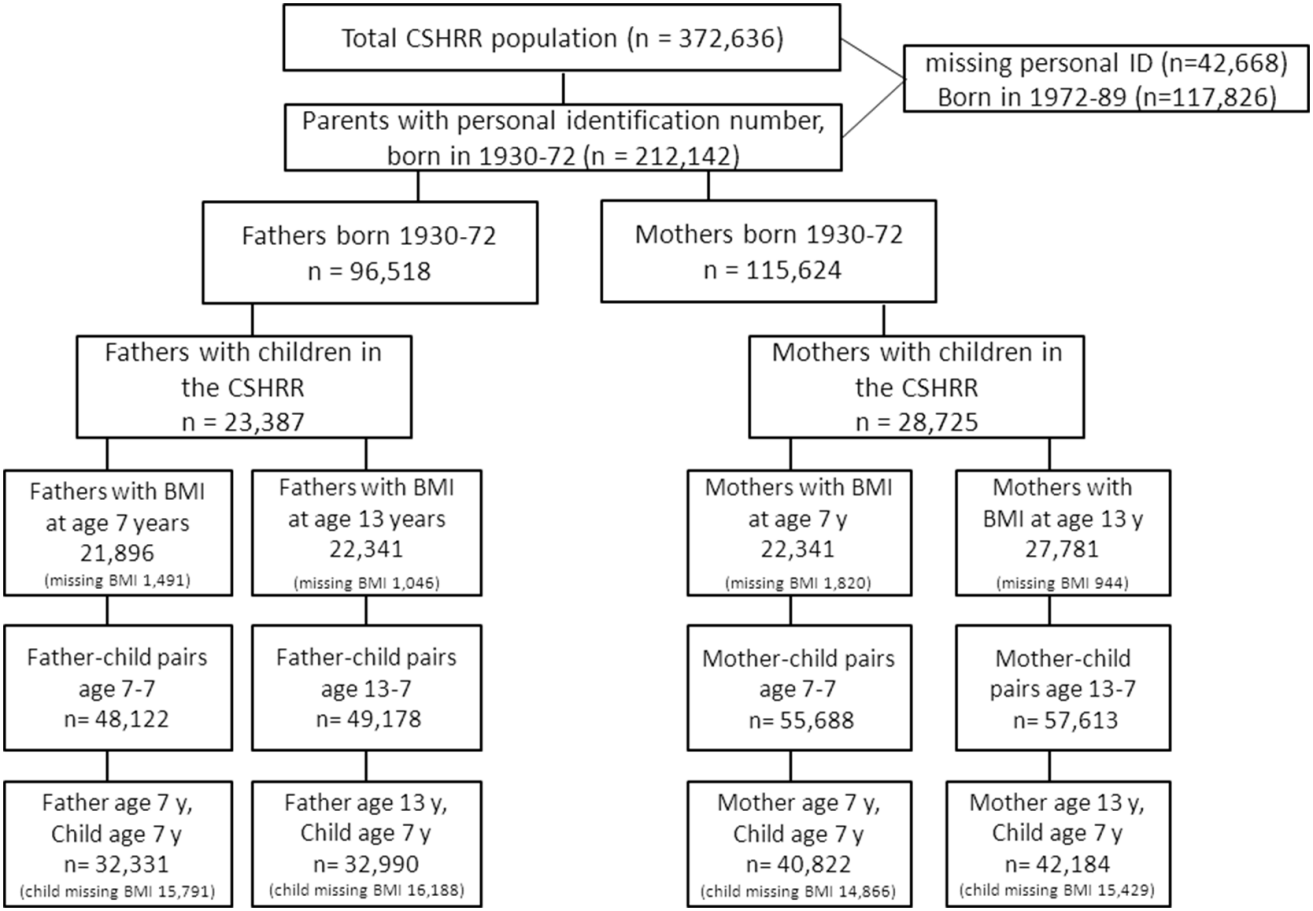
Flow-chart of the study population.

To be eligible for the present study, individuals were required to have a personal identification number, and mothers and fathers had to be born from 1930 through 1972 and to have one or more children born from 1952 through 1989, which would allow children to be located in the CSHRR. Children had to be born after 1951 because, as explained above, the Fertility Register does not consistently provide parental connections for individuals born before this time. To be included in the study, the parents were required to have a measurement of weight and height at 7 or 13 years of age, and the children were required to have these measurements at 7 years of age (the changes in measurement frequency in the schools after 1984 implied that too few in the register have measurement at age 13 years to be included in the present study). Of 212,142 identified parents in the CSHRR, 28,725 mothers and 23,387 fathers fulfilled these criteria. The study population with BMI measures available consisted of 40,822 and 42,184 mother-child pairs at the ages 7–7 and 13–7 years, respectively, as well as 32,334 and 32,990 father-child pairs at the ages 7–7 and 13–7 year (Figure 1).

### Calculation of Body Mass Index

BMI was calculated from the height and weight measurements for parents at ages 7 and 13 years and in the children from the 7-year measurements. Sex- and age-specific BMI z-scores were generated using internal reference populations of boys and girls born during the years 1955–1960 where the prevalence of overweight was low and stable [23]. BMI z-scores were calculated by subtracting the mean BMI in the reference population from the individual’s BMI and then dividing it by the standard deviation in the reference population. If the measurements were taken at the exact age this z-score was used. Otherwise, the z-scores were interpolated to the exact ages of 7 and 13 years if two measurements between the ages 6–8 or 12–14 years, respectively, were available, or extrapolated (z-score carried forward or backward) if only one measurement in the corresponding age interval was available [23].

### Sex-specific secular trends

In accordance with the phases in the development of the obesity epidemic in this population [18], the estimation of parent-child correlations were performed within eight birth year intervals (1952–56, 1957–60, 1961–65, 1966–70, 1971–75, 1976–80, 1981–85, and 1986–89) of the children.

Previous studies have suggested differences between mother-child and father-child correlations in BMI [8], [24], [25], and greater same- than cross-sex intergenerational correlations have been also observed, but the evidence remains ambiguous [24]. Whereas thorough investigation of such differences was not the aim of the present study, we estimated the trends in the intergenerational correlations for each sex of parents and children separately.

### Statistical analyses

All analyses have been performed using Stata 12.0 (StataCorp LP, college Station, Texas; www.stata.com).

The BMI distributions of parents and children including means, standard deviations, medians, 5^th^ and 95^th^ percentiles, were summarized by the eight child birth year intervals. Differences in mean levels across time were tested by linear regression. The BMI distribution of the subset of the CSHRR population used in the present study was validated through a comparison with the BMI distribution in the total CSHRR.

Mother-son and mother-daughter, father-son and father-daughter correlations were estimated by use of BMI values at age 7 years for both generations (7–7 years) as well as by parental BMI values at age 13 years and child BMI values at age 7 years (13–7 years).

Correlation coefficients were estimated for each of the eight birth year intervals. To account for correlations between siblings, robust standard errors for estimates were calculated, using the *vce-cluster command* in Stata throughout analyses. Trends in correlations across time were estimated with linear regression analyses using sex-, age- and birth interval standardized BMI-z scores. Trends in the intergenerational correlations were therefore tested by inclusion of a time variable as well as an interaction term between time and the mother’s or the father’s BMI z-score in these linear regression models, where the BMI z-scores of the children were the response variable. The time variable was numerical ranging from 0 through 37 indicating years since study start. Estimates of changes in correlations were given as changes per birth year of the children with 95% confidence intervals (CI) and exact p-values. Partial correlation analyses with adjustment for parental age at child birth as well as with inclusion of the other parents BMI were conducted and the results are presented in File S1.

### Ethical statement

The person identification numbers were anonymized in the working files by Statistics Denmark, and hence not accessible to the data analyst (T.A.). The Danish Data Protection Agency approved the use of the cohort for this study (according to the Danish law, ethical approval and informed consent is not needed for purely register-based studies).

## Results

The secular trends in mean, standard deviation, median, 5^th^ and 95^th^ percentiles of BMI among parents and their sons and daughters are presented in Table 1. While the mean BMI only increased marginally, the 95^th^ BMI percentiles in the 7 year old children increased steadily from 17.5 kg/m^2^ in the years 1952–56 to 19.8 kg/m^2^ in the years 1985–89, which is reflected in a general BMI increase by 0.12 BMI units (95% CI 0.11–0.13) per birth year interval in both sons and daughters. In comparison, parental mean BMIs were virtually stable across time accompanied by slight increases in SD and upper percentiles. The mean values rose in mothers by 0.01 (0.001–0.020; p<0.001) and in fathers by 0.01 (0.00–0.010; p = 0.08) BMI units per birth year interval. Comparison of BMIs between the included parent-child population and the total CSHRR population of mothers, fathers, and children were made, and no differences in mean and standard deviations of BMIs were observed (Table S1 in File S1). Parental age at child birth increased steadily across time from mean (SD) 21.4 (2.3) to 27.8 (5.1) in mothers and from 22.3 (2.4) to 31.0 (6.2) in fathers, across the entire period, Table 1.

**Table 1:** Show BMI distributions, including mean, standard deviation (SDs), median, and 5^th^ and 95^th^ percentile of mothers at age 7 and 13 years and their sons and daughters at age 7 years by eight intervals of children’s birth year.

	*Child birth year*															
	*1952-56*		*1957-60*		*1961-65*		*1966-70*		*1971-75*		*1976-80*		*1981-85*		*1986-89*	
*Mothers BMIs age 7*	*n* = 2,189		*n* = 4,638		*n* = 7,524		*n* = 7,807		*n* = 6,846		*n* = 4,702		*n* = 4,021		*n* = 3,095	
mean ±SD	15.3	±1.1	15.5	±1.3	15.2	±1.2	15.5	±1.4	15.4	±1.4	15.5	±1.5	15.4	±1.5	15.4	±1.5
median	15.1		15.2		15.3		15.3		15.2		15.3		15.2		15.3	
5th BMI percentile	13.5		13.7		13.6		13.5		13.4		13.4		13.4		13.3	
95th BMI percentile	17.3		17.5		17.6		17.8		17.9		18.0		18.2		17.9	
*BMI age 7 in sons of mothers*	*n* = 1,628		*n* = 2,800		*n* = 4,122		*n* = 4,166		*n* = 3,610		*n* = 2,504		*n* = 2,219		*n* = 1,705	
mean ±SD	15.5	±1.2	15.5	±1.3	15.5	±1.3	15.7	±1.3	15.8	±1.4	16.0	±1.5	16.0	±1.6	16.2	±1.8
median	15.4		15.4		15.4		15.5		15.7		15.8		15.7		15.9	
5th BMI percentile	13.8		13.8		13.7		13.7		13.9		14.0		13.8		13.9	
95th BMI percentile	17.5		17.7		17.8		18.0		18.3		18.8		18.8		19.7	
*BMI age 7 in daughters of mothers*	*n* = 803		*n* = 2,377		*n* = 4,028		*n* = 4,063		*n* = 3,504		*n* = 2,398		*n* = 2,032		*n* = 1,635	
mean ±SD	15.4	±1.5	15.4	±1.4	15.4	±1.5	15.5	±1.5	15.7	±1.6	16.0	±1.7	16.0	±1.8	16.1	±2.0
median	15.3		15.2		15.3		15.3		15.5		15.7		15.8		15.8	
5th BMI percentile	13.4		13.4		13.4		13.5		13.6		13.7		13.7		13.7	
95th BMI percentile	18.1		18.0		18.2		18.3		18.6		19.1		19.3		19.7	
Mother's age when giving birth, mean±SD	21.4	±2.3	22.9	±3.2	23.7	±4.1	24.4	±4.5	25.2	±4.5	25.9	±4.9	26.8	±5.0	27.8	±5.1
*Fathers BMIs age 7*	*n* = 977		*n* = 2,752		*n* = 5,253		*n* = 6,080		*n* = 6,052		*n* = 4,466		*n* = 3,764		*n* = 2,987	
mean ±SD	15.4	±1.1	15.4	±1.1	15.5	±1.1	15.6	±1.1	15.5	±1.2	15.5	±1.2	15.5	±1.2	15.5	±1.3
median	15.3		15.3		15.4		15.5		15.4		15.4		15.4		15.4	
5th BMI percentile	13.9		13.8		13.9		13.9		13.9		13.8		13.7		13.7	
95th BMI percentile	17.2		17.3		17.4		17.5		17.5		17.5		17.5		17.6	
*BMI age 7 in sons of fathers*	n = 720		*n* = 1,617		*n* = 2,884		*n* = 3,235		*n* = 3,197		*n* = 2,387		*n* = 2,088		*n* = 1,661	
mean ±SD	15.4	±1.2	15.5	±1.2	15.5	±1.3	15.6	±1.3	15.8	±1.4	16.0	±1.5	16.0	±1.6	16.2	±1.8
median	15.3		15.4		15.4		15.5		15.7		15.8		15.7		15.8	
5th BMI percentile	13.7		13.7		13.6		13.8		13.9		14.0		13.8		13.9	
95th BMI percentile	17.4		17.6		17.7		17.9		18.3		18.7		18.8		19.8	
*BMI age 7 in daughters of fathers*	*n* = 357		*n* = 1,386		*n* = 2,790		*n* = 3,189		*n* = 3,185		*n* = 2,286		*n* = 1,905		*n* = 1,642	
mean ±SD	15.4	±1.5	15.4	±1.4	15.4	±1.5	15.5	±1.5	15.7	±1.5	15.9	±1.7	16.0	±1.8	16.1	±1.9
median	15.2		15.2		15.2		15.3		15.5		15.7		15.7		15.7	
5th BMI percentile	13.4		13.5		13.4		13.5		13.7		13.7		13.7		13.7	
95th BMI percentile	17.7		18.0		18.0		18.2		18.6		18.9		19.2		19.8	
Father’s age, mean ±SD	22.3	±2.4	24.2	±3.0	25.4	±4.0	26.3	±4.6	27.4	±4.8	28.6	±5.4	29.7	±5.8	31.0	±6.2

**Figure 2:**
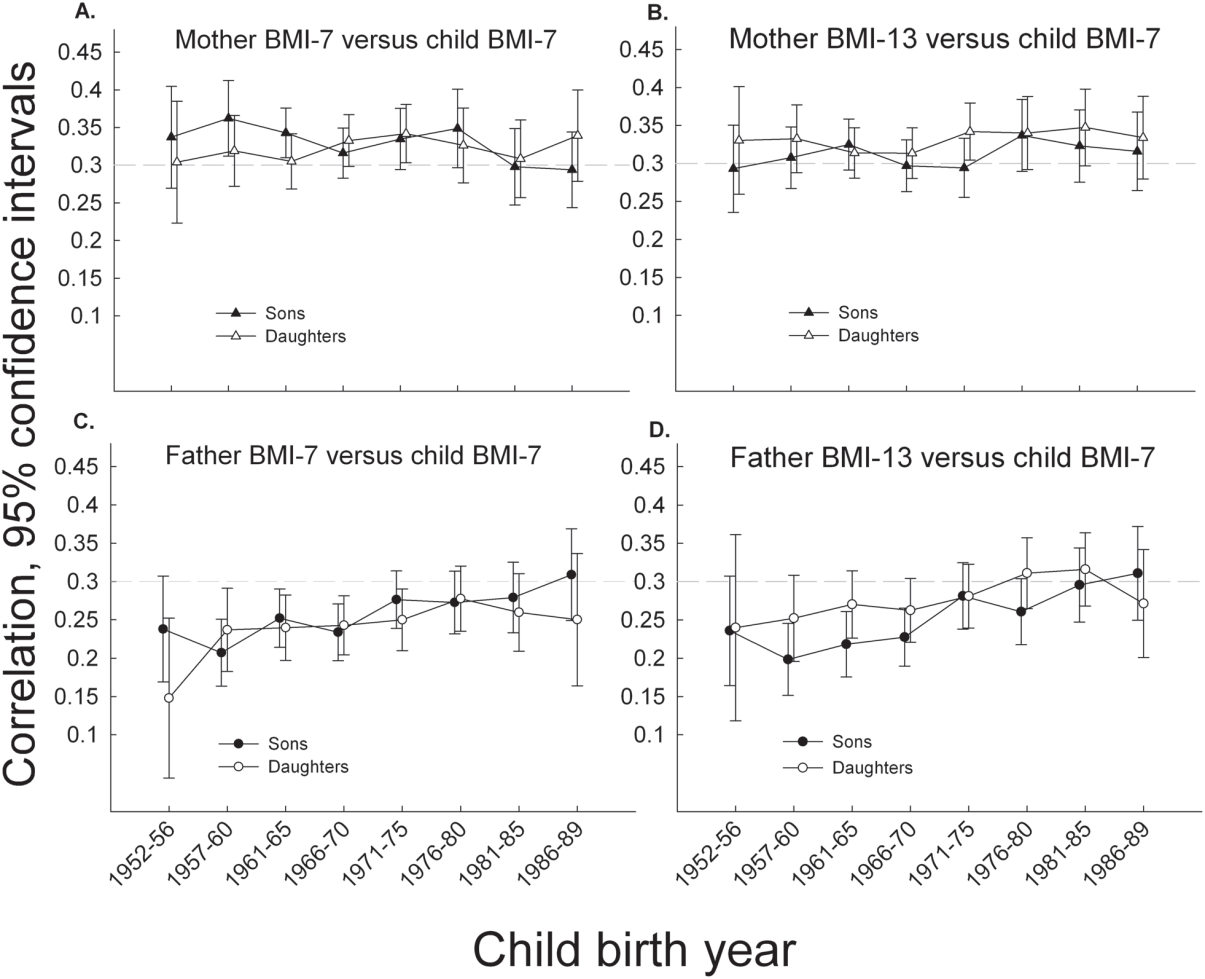
The figure shows four panels of BMI correlations across eight intervals of child birth year. Mother-child correlations shown in panels A & B and father-child correlations shown in panels C & D using age 7 and 13 year BMI z-scores (BMI-7 and BMI-13, respectively), respectively, in parents, and age 7 year BMI z-scores (BMI-7) in children. Test for trends across birth year intervals: Mother BMI-7 versus son BMI-7, p = 0.006; mother BMI-13 versus daughter BMI-7, p = 0.84; father BMI-7 versus son BMI-7, p = 0.007; father BMI-7 versus daughter BMI-7, p = 0.37. Mother BMI-13 versus son BMI-7, p = 0.96; mother BMI-13 versus daughter BMI-7, p = 0.56; father BMI-13 versus sonBMI-7, p<0.001; father BMI-13 versus daughter BMI-7, p = 0.37. The grey dotted line is a grid line at the correlation of 0.30.

During the study period, the mother-child correlations in BMI were rather stable. The correlations between mothers and sons ranged from 0.29–0.36 and they decreased marginally, albeit significantly across time at ages 7–7 years (−0.002/year, CI −0.003 to 0.000, p = 0.006) (Figure 2A), whereas those at 13–7 years remained stable (<0.0004/year, CI −0–001 to 0.001, p = 0.96) (Figure 2B).

Mother-daughter correlations ranged from 0.30–0.34, and they were stable at ages 7–7 years (0.0001/year, CI −0.001 to 0.002, p = 0.84) (Figure 2A), and at 13–7 years (0.0004/year, CI −0.001 to 0.002, p = 0.56) (Figure 2B).

In contrast, father-child correlations in BMI showed increasing trends. Thus, the father-son correlations increased significantly during this period, both at ages 7–7 years (0.002/year, CI 0.001 to 0.004, p = 0.007) (Figure 2C), and at ages 13–7 years (0.003/year, CI 0.001 to 0.005, p<0.001). The increase in father-daughter correlations were, however, insignificant both at ages 7–7 years (0.001/year, CI −0.001 to 0.002, p = 0.37) (Figure 2C), and at ages 13–7 years (0.001/year, CI −0.001 to 0.003, p = 0.18).

Inclusion of parental age at child birth through partial correlation analyses only changed correlation estimates marginally (Table S2 in File S1). Additional inclusion of mother’s BMI in analyses of father-child pairs lead to increases in the correlation estimates, whereas mother-child correlations remained stable when paternal BMI and the age of parents at the child’s birth were included (Figure S1 in File S1).

## Discussion

In this study we investigated changes in parent-child correlations of childhood BMIs in two generations during the development of the obesity epidemic in a Danish population. The mother-child correlations did not show any significant trends between birth years 1952 through 1989 barring a slight decreasing trend in the correlations between mother-son pairs at ages 7–7 years. In contrast, the father-child correlations increased across time, significantly for the father-son correlations, although not for the father-daughter correlations. The father-child correlations may, however be influenced by the registration process of fathers back in time, especially in the earliest cohorts, which may have lowered these correlation estimates.

In general, the level of the correlations estimated in the present study was fairly comparable to the few other studies of intergenerational correlations of BMI measures from the same ages, although none of them compared the correlations between different birth cohorts. An investigation in the 1958 British Birth Cohort investigated correlations of childhood BMI measures in two generations of females (4–8 years) [9], and found intergenerational correlation estimates between 0.21 and 0.28 [9]. In the present study, in the same birth cohort interval, i.e. 1986–89, correlations of 0.35 between mother-daughter pairs were found. The difference in the estimates may be due to a different, wider, and younger age span of the daughters in the British study as compared with ours [5], [24]. In a study by Johnson et al., based on 1,443 fathers and children with measured adult heights and weights from the 1970s [5], father-child correlation were estimated as 0.23, which is comparable to the estimate of 0.25 from the present study.

Some studies have shown a greater mother-child than father-child BMI resemblance [5], [25]. However, not all studies support such effects even though it is plausible that specific maternal effects may make mother-child correlations greater than father-child correlations [2], [5], [6], [26], [27]. Valid comparisons of these correlations would require elimination of non-biological father-child pairs from the correlation analyses. The present data does not allow complete or comparable identification of biological parent-child relationships, so we did not aim to examine differences between mother-child and father-child resemblance, but only to investigate changes in mothers or fathers influence on their children’s BMIs across time. Mother-child resemblance was essentially stable across time and the increasing trends in the father-child correlations were rather small and likely due to changes in assignment of non-biological fathers in the registration system. Thus, the results indicate that the environmental influences driving the obesity epidemic has not operated through changes in the parent-child relationships expressed in their childhood BMI. It is possible that trends in the parent-child correlations have been masked by random influences from the obesogenic environment on the trait in either generation. However, such effect would have been expected also to operate among adults, and it was not seen in previous studies of adult twins and brother pairs [14]–[16].

The finding of no such trends does not preclude that there could have been various changes in the modifiable determinants of the parent-child correlations, possibly outbalancing each other. Thus, specific maternal effects, induced by perinatal programming or by the common household environment provided mostly by the mothers, could have changed [3], [8], [24], [25]. Moreover, changes in shared environment, such as an increase in influence from fathers, in epigenetic influences [28]–[32] or in non-paternity [33], [34] may have influenced the father-child correlations to increase.

Although the twin and adoption studies suggest that the shared environment in childhood has little persisting influence on family resemblance when the family members do not share household anymore [11], [13], [35], such effects may still be operative [16], [36]. Changes in family lifestyles have occurred along with the changes in the workforce [37]. As women entered the labor market [38], changes in the influence from fathers on children’s BMIs may have occurred, but not necessarily at the direct expense of the resemblance with mothers. While recognizing that there might be true differences in trends between maternal and paternal correlations with their children and between paternal correlations with sons and daughters, speculations about the reasons for such differences should be postponed until data without the uncertainty regarding the registration of the biological father are available.

Through partial correlations the influence from parental age at child birth were investigated, but it did not show influence on the correlation estimates. As BMI increases with age, higher age at conception may be expected to influence the intergenerational correlations if pre-conceptional, or intrauterine programming effects exists. While we did not have adult BMIs available, age was used as a surrogate measure of possible BMI-increase with age, but it may not be able to capture such effect.

Assortative marriages by high BMI percentiles have previously been observed in a married subpopulation of the CSHRR, and the increases in assortative marriages in the cohorts from the 1970s and onwards were distinct and significant [20]. However, assortative marriages were not observed in the central or lower parts of the BMI distribution, diminishing the possibility of influencing the parent-child correlation across the whole range of BMI. Inclusion of the other parents BMI through partial correlations showed no correlation change between mother-child pairs, whereas the father-child correlations strengthened slightly.

Our study has both strengths and limitations. The validity is high due to the prospectively measured heights and weights on all children attending the schools in the Copenhagen municipality with limited risk of measurement and selection bias. Recording of the family relationships through personal identification numbers became routine from 1968, so the biological parent-child connections may not be complete and correct throughout the study period, which may have biased especially the father-child correlations downwards in the early period, but the correlations for children born during the last part of the period are likely to be close to true biological father-child relations apart from hidden non-paternity. There are no reasons to believe that the mother-child correlations were notably affected by the registration procedures. Inclusion of family members in the present study was only possible if both generations had attended a school in the municipality of Copenhagen, and the study population size was reduced due to families moving out of the Copenhagen region during the 1960s and 1970s to newly developed suburbs [38]. However, a comparison of the included sample with the original larger CSHRR showed no differences in BMI distributions (Table S1 in File S1). One potentially important limitation of the present study is that the parental generations BMIs were measured before the second rapid phase of obesity epidemic in this population. Thus, although the parents were exposed to the first phase of the obesity epidemic with increases in the upper percentiles and in the standard deviation of their BMI values, there were only very small increases in mean and median BMIs in childhood of the parents during the study period. Investigation of trends in familial resemblance in more recent birth cohorts, where distinct increases in childhood BMIs of parents have taken place, are needed to address this effect.

Whereas this study indicates that there is no convincing relationship between the general familial resemblance in childhood BMI between parents and children and the obesity epidemic, investigations of the familiar resemblance in different segments of the population defined by parental BMI, urban versus rural residence, educational level, and socio-economic position may reveal differences in segment-specific correlations.

The take-off of the obesity epidemic in this population was not reflected in accompanied changes in the intergenerational BMI correlations. Father-child resemblance increased slightly, possibly explained through changes in the family relationships, whereas mother-child resemblance in childhood BMIs were stable across time.

## Supporting Information

File S1 Figure S1, The figure show four panels of parent-child BMI correlations adjusted for parental age at child birth as well as for the other parents BMI through partial correlations. Mother-child correlations are shown in panels A & B and father-child correlations are shown in panels C & D using age 7 and 13 year BMI z-scores (BMI-7 and BMI-13, respectively), respectively, in parents, and age 7 year BMI z-scores (BMI-7) in children. Test for trends across birth year intervals, performed by inclusion of a product term between time and birth interval standardized residuals: Mother BMI-7 versus son BMI-7, p = 0.699; mother BMI-7 versus daughter BMI-7, p = 0.902; father BMI-7 versus son BMI-7, p = 0.007; father BMI-7 versus daughter BMI-7, p = 0.37. Mother BMI-13 versus son BMI-7, p = 0.96; mother BMI-13 versus daughter BMI-7, p = 0.56; father BMI-13 versus son BMI-7, p<0.001; father BMI-13 versus daughter BMI-7, p = 0.37. The grey dotted line is a gridline given at correlation coefficient 0.3. Table S1, The table shows child BMI (mean± SD) at age 7 years in the included mother-child and father-child populations (with parental BMIs available either at age 7 or at age 13 years) as well as in the rest of the Copenhagen school health record register (CSHRR) of children from the same birth years. Table S2, Show results from partial correlations of mother-child or father-child BMI z-scores at ages 7–7 or 13–7 years, respectively, adjusted for mothers or fathers age at child birth.
(DOCX)
