# Supplementary material for: Trends in Parent-Child Correlations of Childhood Body Mass Index during the Development of the Obesity Epidemic
Source: PLoS One. 2014 Oct 17;9(10):e109932. doi: 10.1371/journal.pone.0109932 (PMC4201474; doi:10.1371/journal.pone.0109932)
Supplement: File S1 — Figure S1, The figure show four panels of parent-child BMI correlations adjusted for parental age at child birth as well as for the other parents BMI through partial correlations. Mother-child correlations are shown in panels A & B and father-child correlations are shown in panels C & D using age 7 and 13 year BMI z-scores (BMI-7 and BMI-13, respectively), respectively, in parents, and age 7 year BMI z-scores (BMI-7) in children. Test for trends across birth year intervals, performed by inclusion of a product term between time and birth interval standardized residuals: Mother BMI-7 versus son BMI-7, p = 0.699; mother BMI-7 versus daughter BMI-7, p = 0.902; father BMI-7 versus son BMI-7, p = 0.007; father BMI-7 versus daughter BMI-7, p = 0.37. Mother BMI-13 versus son BMI-7, p = 0.96; mother BMI-13 versus daughter BMI-7, p = 0.56; father BMI-13 versus son BMI-7, p<0.001; father BMI-13 versus daughter BMI-7, p = 0.37. The grey dotted line is a gridline given at correlation coefficient 0.3. Table S1, The table shows child BMI (mean± SD) at age 7 years in the included mother-child and father-child populations (with parental BMIs available either at age 7 or at age 13 years) as well as in the rest of the Copenhagen school health record register (CSHRR) of children from the same birth years. Table S2, Show results from partial correlations of mother-child or father-child BMI z-scores at ages 7–7 or 13–7 years, respectively, adjusted for mothers or fathers age at child birth. (DOCX) [file pone.0109932.s001.docx]

**Supporting Information**

**Supplementary Table S1** The table shows child BMI (mean± SD) at age 7 years in the included mother-child and father-child populations (with parental BMIs available either at age 7 or at age 13 years) as well as in the rest of the Copenhagen school health record register (CSHRR) of children from the same birth years.

**Supplementary Table S2** Show results from partial correlations (r) of mother-child or father-child BMI z-scores at ages 7-7 or 13-7 years, respectively, adjusted for mothers or fathers age at child birth.

**Supplementary Figure S1** The figure show four panels of parent-child BMI correlations adjusted for parental age at child birth as well as for the other parents BMI through partial correlations. Mother-child correlations are shown in panels A & B and father-child correlations are shown in panels C & D using age 7 and 13 year BMI z-scores (BMI-7 and BMI-13, respectively), respectively, in parents, and age 7 year BMI z-scores (BMI-7) in children. Test for trends across birth year intervals, performed by inclusion of a product term between time and birth interval standardized residuals: Mother BMI-7 versus son BMI-7, p=0.699; mother BMI-7 versus daughter BMI-7, p=0.902; father BMI-7 versus son BMI-7, p=0.007; father BMI-7 versus daughter BMI-7, p=0.37. Mother BMI-13 versus son BMI-7, p=0.96; mother BMI-13 versus daughter BMI-7, p=0.56; father BMI-13 versus son BMI-7, p<0.001; father BMI-13 versus daughter BMI-7, p=0.37. The grey dotted line is a gridline given at a correlation coefficient of 0.3.
